# Percutaneous Mechanical Aspiration for Right-Sided Endocarditis: A Step Forward While Awaiting Randomized Data

**DOI:** 10.1016/j.jscai.2026.105569

**Published:** 2026-08-04

**Authors:** Pedro Engel Gonzalez, Siavash Saadat

**Affiliations:** a Division of Cardiology, Department of Medicine, Henry Ford Health, Detroit, Michigan; b Department of Surgery, UMass Chan Medical School – Baystate, Springfield, Massachusetts

**Keywords:** aspiration, endocarditis, infective endocarditis, percutaneous mechanical aspiration

Traditionally, the treatment for infective endocarditis (IE) has consisted of prompt diagnosis, appropriate antibiotic therapy, and early surgical intervention for patients presenting with valve dysfunction resulting in heart failure, persistent infection, abscess, heart block, highly resistant organisms, or recurrent emboli with persistent vegetations. This group of indications comprises the only class I recommendations for intervention in the latest iteration of the American Heart Association/American College of Cardiology Valvular Heart Disease guidelines.^1^ In fact, historical data demonstrate a 21% in-hospital mortality rate among patients with IE and heart failure managed surgically compared with 45% for those treated medically.

In real-world practice, however, many of these patients are elderly, carry a high burden of comorbidities, and frequently present with hemodynamic compromise—rendering them poor or prohibitive surgical candidates. Consequently, it is estimated that surgical management is ultimately performed in only 5% to 40% of eligible patients.^2^ Furthermore, tricuspid valve surgery for right-sided IE carries a reported operative mortality of 6% to 10%.

Driven by this unmet clinical need, large-bore transcatheter aspiration systems have gained Food and Drug Administration clearance for debulking right-sided intracardiac masses. These include systems powered by extracorporeal membrane oxygenation circuit connections, such as AngioVac (AngioDynamics), circuit-connected systems like the Penumbra Lightning 12 or 16 (Penumbra), and vacuum-assisted manual aspiration systems like AlphaVac (AngioDynamics). Over the past several years, these platforms have been increasingly utilized for tricuspid valve endocarditis with large vegetations; however, current evidence supporting transcatheter therapies for this indication has remained largely limited to isolated case reports and small, single-center series.

The manuscript by Robinson et al^3^ in this issue of *JSCAI* addresses this gap. The authors present a multicenter, propensity-matched analysis evaluating outcomes of percutaneous mechanical aspiration across 19 US tertiary centers. The investigators are to be commended for this first large-scale effort to acquire critical observational data regarding the feasibility, safety, and—to some extent—efficacy of this approach.

The Cardiac Lesion Extraction and Aspiration Registry for Infective Endocarditis (CLEAR-IE) registry takes a meaningful step forward by comparing outcomes between devices grouped by their aspiration mechanics: continuous flow (CF) systems versus noncontinuous flow (NCF) systems. In their propensity-matched analysis, procedural success—defined as a ≥70% reduction in vegetation size or a residual vegetation ≤10 mm on intraprocedural echocardiography—was similar between cohorts; however, the primary safety endpoint (a composite of in-hospital mortality, new pulmonary embolism, urgent surgical intervention, or blood loss >150 mL) was significantly lower in the CF group.

Based on the presented data, this disparity in safety appears driven primarily by higher rates of acute blood loss and subsequent transfusion requirements in the NCF cohort. This finding is mechanically predictable, as NCF systems rely on intermittent suction without the benefit of autologous reinfusion. Notably, the incidence of worsening tricuspid regurgitation was nontrivial in both groups, ranging from 14.7% to 22.9%, without a significant between-group difference. No significant differences were observed regarding in-hospital mortality (ranging from 5.9% to 14.1%) or 6-week mortality (ranging from 9.4% to 20.6%).

Despite these insights, several important limitations restrict the immediate application of these data to routine clinical practice. The primary constraint is the profound sample size imbalance between the 2 groups, with only 36 patients in the NCF cohort compared with 202 patients in the CF cohort. Although the authors attempted to mitigate this discrepancy by utilizing a 3:1 propensity-score matching algorithm, such a marked sample size asymmetry leaves a high probability of residual confounding variables that remain unaccounted for. Compounding this issue, there is no objective or standardized protocol for device selection; however, it does appear that NCF systems were used more commonly in patients with lead-associated vegetations, a finding the authors propose may stem from operator preference for device maneuverability. Similarly, the absence of a standardized protocol for procedural technique, systematic postprocedural imaging, or centralized outcomes adjudication (as endpoints were self-reported by participating sites) further hinders this analysis. Nonetheless, the CLEAR-IE registry represents a vital step in the right direction, offering a clearer descriptive understanding of real-world percutaneous mechanical aspiration utilization across US tertiary centers.

The “elephant in the room,” however, cannot be addressed by a registry of this design. Prior to the widespread proliferation of these devices and the accelerating adoption of percutaneous extraction strategies, it is essential to define exactly how this therapeutic option fits within the current standard of care. As previously noted, the established baseline remains guideline-directed antibiotics and surgical management in indicated scenarios. Crucially, there are currently no randomized controlled trials comparing specific aspiration devices, nor are there trials evaluating percutaneous intervention against medical therapy alone—which remains the default strategy for patients deemed inoperable. More robust, randomized data are urgently required to identify optimal devices, refine procedural strategies, and establish definitive best practices.

## CRediT authorship contribution statement

**Pedro Engel Gonzalez:** Conceptualization, Writing – original draft, Writing – review & editing. **Siavash Saadat:** Writing – original draft, Writing – review & editing.

## Declaration of competing interest

The author declared no potential conflicts of interest with respect to the research, authorship, and/or publication of this article.
